# Transpedicular Endoscopic Surgery for Highly Downmigrated L5-S1 Disc Herniation

**DOI:** 10.1155/2019/5724342

**Published:** 2019-02-25

**Authors:** Guntram Krzok

**Affiliations:** Department of Orthopedic Surgery, SRH Hospital Waltershausen-Friedrichroda, Friedrichroda, Germany

## Abstract

Endoscopic surgery for highly downmigrated disc herniation at level L5-S1 is a challenging technique. Most surgeons prefer the interlaminar access because of the special anatomy of the L5-S1 disc level, i.e., narrow neuroforamen and large interlaminar window. Transforaminal access to the neuroforamen L5-S1 is difficult in cases with high iliac crest. Here, the access to the highly downmigrated disc herniation with the recently reported technique of transpedicular endoscopic surgery by Krzok et al. was described. In 3 cases with highly downmigrated disc herniation of L5-S1, the sequester was removed successfully through the bone hole of S1 pedicle. This technique is demanding for experienced endoscopic surgeons.

## 1. Introduction

Endoscopic spinal surgery has been still in their infancy after the beginning with Hijikata's technique [1] and the first experience of Kambin and Gellman [2] followed by the era of intradiscal techniques [3]. After Hoogland's transforaminal access to spinal canal [4] and the interlaminar endoscopic technique of Ruetten et al. [5], it is possible to perform endoscopic surgery for all kinds of lumbar disc herniation [6].

Most surgeons prefer the interlaminar endoscopic surgery at level L5-S1 [5–9]. The anatomy of L5-S1 differs from the others in the lumbar spine because of the small neuroforamen in contrast to the large interlaminar window. The pedicle S1 part of the canal sacral and the space of L5-root are small. Transforaminal access to the level L5-S1 may be challenging because of the oblique angle in cases with high iliac crest. The narrow neuroforamen must be widened by reaming or drilling combined with endoscopic high-speed drill. Without widening the neuroforamen L5-S1, we do not have enough place for endoscopic discectomy and the L5-root can be damaged.

Yeom et al. [10] preferred the contralateral transforaminal access, but Ahn et al. [11] and Choi et al. [12] the transforaminal access and Hu et al. [13] a combined access through facet and partial pedicle. The endoscopic surgery for highly downmigrated L5-S1 disc herniation is a challenge for the spine surgeons [8, 14, 15]. The sequester must be removed from the sacral canal to decompress the S1 root.

**Figure 1:**
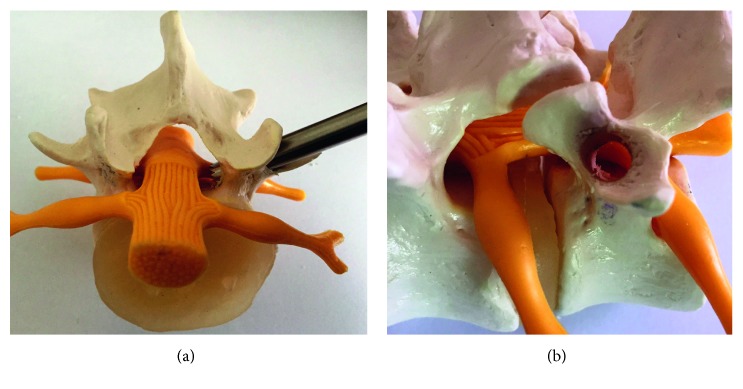
Models of the lumbar spine with transpedicular access parallel to the transverse process and the hole of the pedicle.

Recently, we reported about a new endoscopic technique [16–18] by transpedicular access in cases of highly migrated disc herniation and facet cysts (Figure 1). The technique of Krzok et al. [16–18] has been developed in the last 5 years. Microscopic access to thoracic disc herniations through the pedicle is well known [19], but the anatomy of lumbar pedicle allows only endoscopic surgery over a small bone hole. Case reports of Uniyal et al. [20] and Quillo-Olvera et al. [21] at levels L3-4 and L4-5 confirm our results. In contrast to Uniyal et al. [20], we found no contraindications in the axillary sequester especially in highly upmigrated disc herniation, but the technique for highly upmigrated disc herniation is more difficult. If the size of pedicle is 12 mm or more, we find no contraindications in upper levels. We have done the transpedicular access for far-dislocated sequester at all levels of the lumbar spine including L1-2.

To make a hole through the S1 pedicle, we can choose the access from lateral through the ilium [22] or the transpedicular access (like the transforaminal access but more caudally and medially). We report about three cases with highly downmigrated disc herniation where we used the transpedicular endoscopic technique.

## 2. Case Reports

### 2.1. Operative Procedure

For the transpedicular endoscopic procedure, the patient was positioned laterally with flexed hips and knees, and the procedure was done under local analgesia and intravenous sedation; the level of anesthetic was titrated so the patient was able to communicate with the surgeon throughout the procedure. The joimax® TESSYS endoscopic system with drill technique was used for the procedure. Percutaneous entry was established by advancing through the skin 12 cm lateral to the midline over the iliac crest for the pedicle S1. Using intermittent fluoroscopic guidance, alternating between lateral and anterior-posterior (AP) view, a 25 cm 18-gauge needle was advanced and placed at the lateral pedicle over the transverse process.

After feeling the bone contact with the needle and fluoroscopic view in two planes, the needle can be replaced by a K-wire. A skin cut of depth 6 mm is made, and the soft tissue is dilated by using rods of 4 and 8 mm till the pedicle.

**Figure 2:**
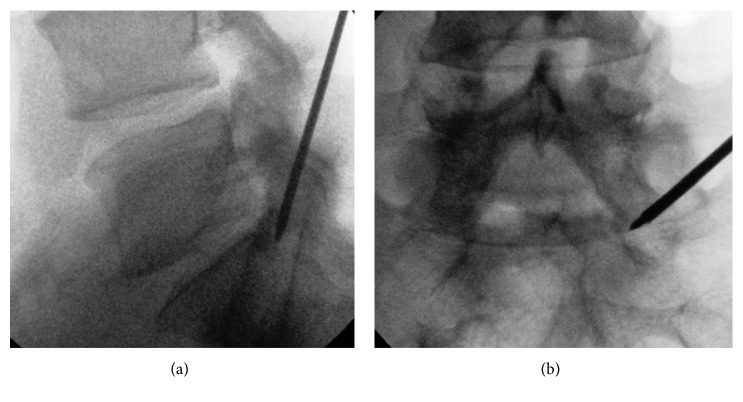
Jamshidi needle entering the S1 pedicle.

With the guidance of K-wire, the Jamshidi needle (Figure 2) was placed on the pedicle and under fluoroscopic view inserted through the bone till the sacral canal. The access point for the bone is the middle portion of the pedicle. The success of the surgery depends on the right puncture at this step. The loss of resistance and sometimes pain in the leg indicate successful puncture. The Jamshidi needle can be replaced by the K-wire and disposable bone drill of 4 mm.

**Figure 3:**
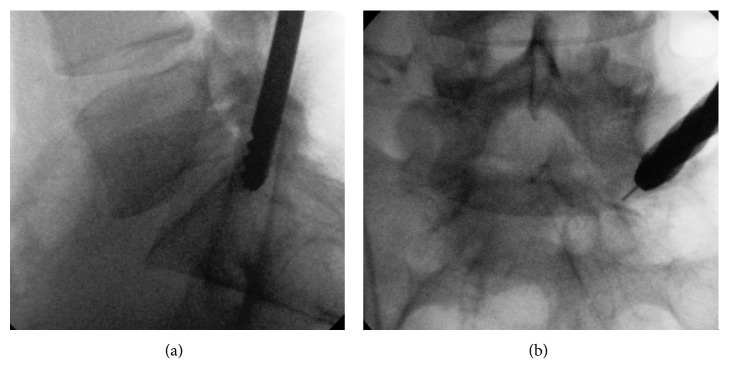
Increasing the bone hole by using 8 mm drills.

**Figure 4 fig4:**
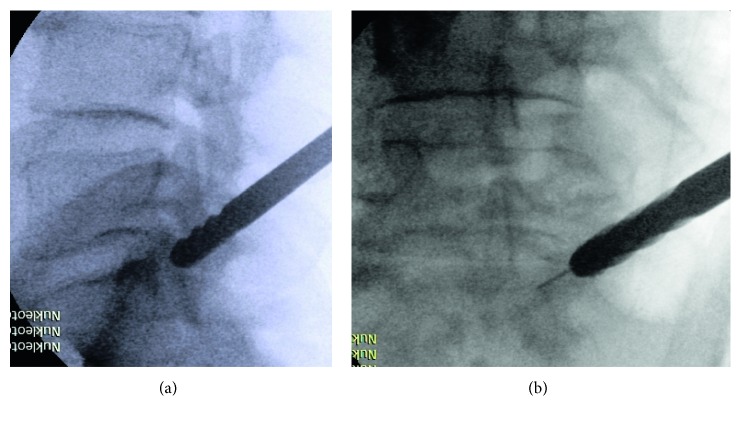
Bone drill through the S1 pedicle.

**Figure 5:**
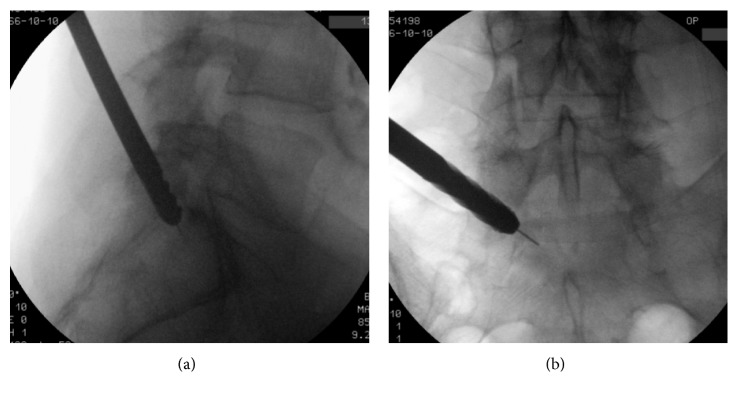
Bone drills through the S1 pedicle.

**Figure 6:**
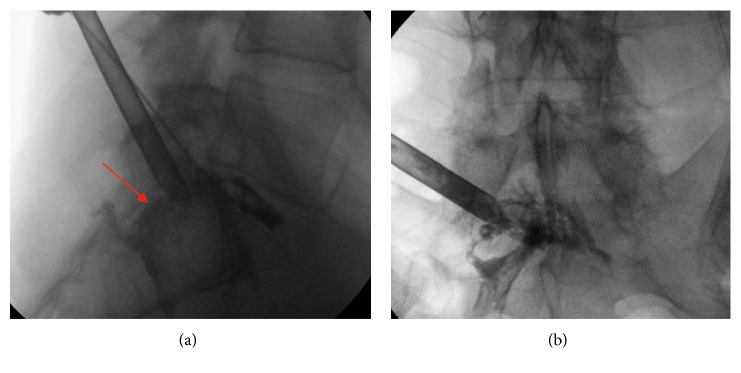
Working tube inside the hole of the S1 pedicle with discography over extra puncture.

Now, a small hole made in the bone should be increased step by step with drills of increasing diameter from 5 to 8 mm using K-wire guidance (Figures 3 [Fig fig4]–5). After making a hole of 8 mm through the pedicle, we insert a working tube (Figure 6). Before endoscopy, we made an additional puncture on the disk above the pedicle with a mixture of contrast medium (Solutrast® (3ccl) and Methylene blue 0 (1ccl)). At last, we insert the endoscope (joimax® GmbH) with a working channel of 3.8 mm.

**Figure 7:**
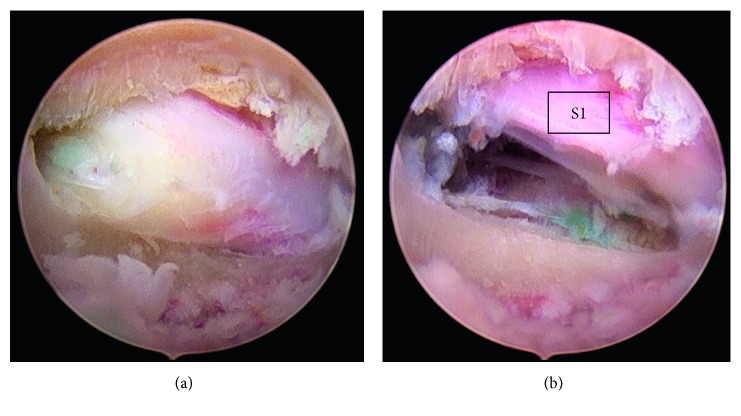
Endoscopic view to the pedicle hole with sequester and after decompressing S1 root.

**Figure 8 fig8:**
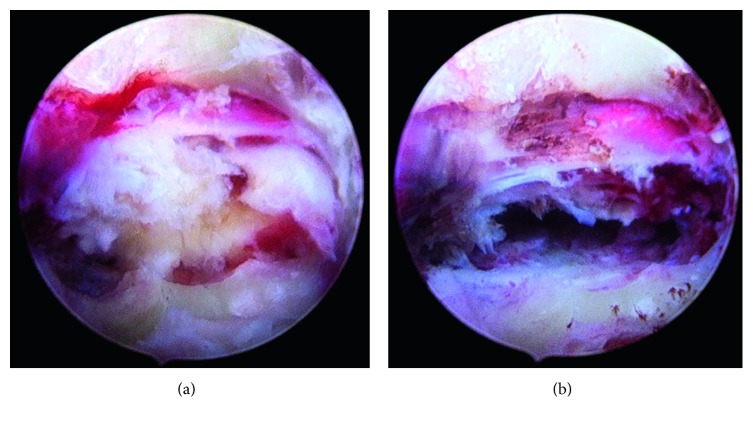
Endoscopic view of the sequester inside the sacral canal and after removing and decompressing the S1 root.

**Figure 9:**
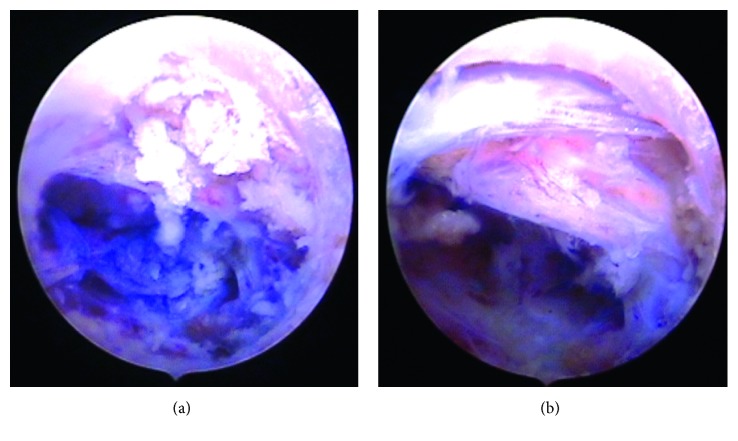
Endoscopic view to the pedicle hole of S1 with blue-stained sequester and after removing the sequester.

Under endoscopic view, we removed some bone fragments, and after cleaning with the radiofrequency probe, we could see blue-stained sequester under the S1 root (Figures 7(a) [Fig fig8]–9(a)). In all cases, we used an additional bone shaver system (“Shrill” from joimax® GmbH) to get a perfect access to the sequester. With the help of flexible forceps, the sequester could be carefully removed.

Successful decompression made a free floating of root and epidural space (Figures 7 [Fig fig8]–9).

### 2.2. Study

We used the new technique in 3 men (Table 1). In all men, the herniation was left-sided.

The preoperative pain score for leg pain was between 7 and 8.5 (on average 7.8). In 2 cases, weakness in foot flexion was observed (M3 and M3-4).

The drill technique combined with the bone shaver was used in all cases.

**Figure 10:**
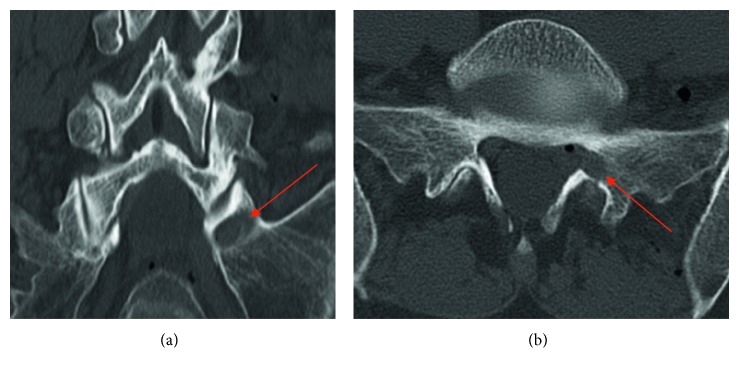
Lumbar CT 1 day after surgery of case 3 with bone hole of the pedicle.

**Figure 11:**
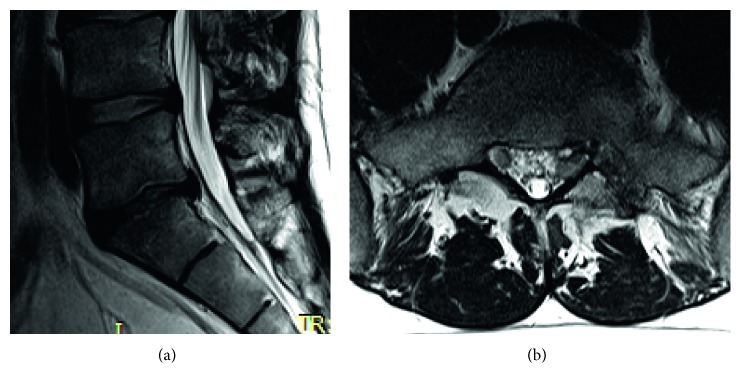
Lumbar MRI of case 1, 4 weeks after surgery.

**Figure 12:**
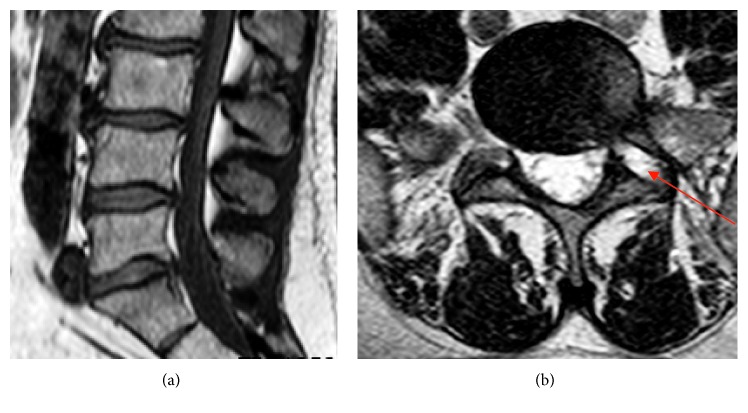
Postoperative lumbar MRI with completely removed sequester and bone hole through the pedicle.

Postoperative CT scan was performed 1 day after operation (Figure 10), and MRI scan was performed 4 weeks after surgery (Figures 11 and 12).

### 2.2.1. Case 1

**Figure 13:**
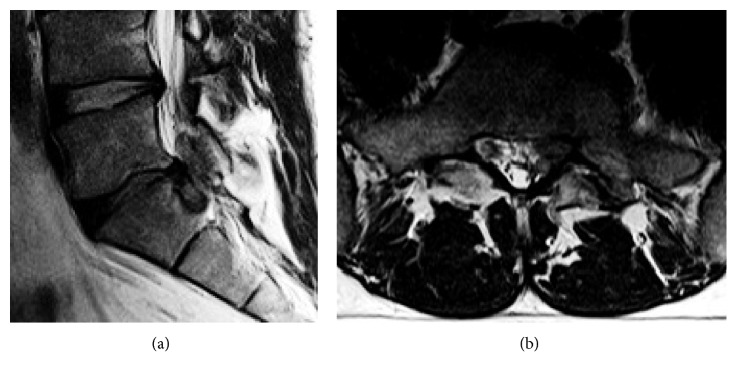
Lumbar MRI of case 1 showing highly migrated disc L5-S1 herniation inside the sacral canal.

The 31-year-old male suffered from severe pain in the left leg dorsally till fifth toe since 3 months. The VAS for low back pain was 3-4, and for leg pain, VAS was 8. Periradicular injections and physiotherapy were unsuccessful. Physical examination revealed positive straight leg test at 20°, absence of Axilles tendon reflex, and weakness of foot extension (M3-4). The MRI of the lumbar spine (Figure 13) showed a large highly caudally migrated disc herniation in the left side. The sequester was inside the canal sacral with left side S1 compression. After surgery, there was a rapid recovery. The VAS for leg pain was reduced to 2 the next day. The straight leg test was negative. The weakness improved to M4, and he completely recovered after 6 weeks. Postoperative MRI showed a good decompression with a very small rest disc piece inside the canal sacral (Figure 11).

### 2.2.2. Case 2

**Figure 14:**
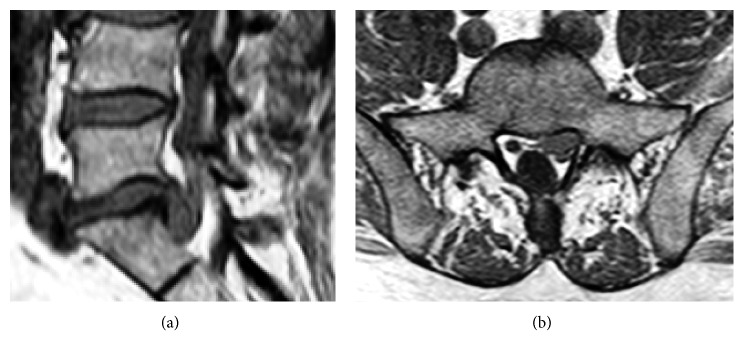
Lumbar MRI of case 2 with large left side caudally dislocated herniation.

The 56-year-old male suffered from pain (VAS 8-9) in the left leg for two weeks. The straight leg test was positive at 40°. No weakness, but numbness at the left side of leg and plantar foot was observed. The MRI scan of the lumbar spine (Figure 14) revealed a huge highly caudally migrated herniation with left side compression of S1. We performed the surgery through the pedicle S1 left-side (Figure 4). Through the bone hole of the S1 pedicle, we found a large sequester (Figure 8(a)); and after removing the sequester by flexible forceps, the S1 root was found floating freely inside the canal (Figure 8(b)).

The leg pain disappeared (VAS 2) immediately, but the numbness disappeared after 4 weeks. Control MRI (Figure 12) showed a complete removal of the sequester and relieved S1 root after 4 weeks. The access way through the S1 pedicle is also shown up (red arrow).

### 2.2.3. Case 3

**Figure 15:**
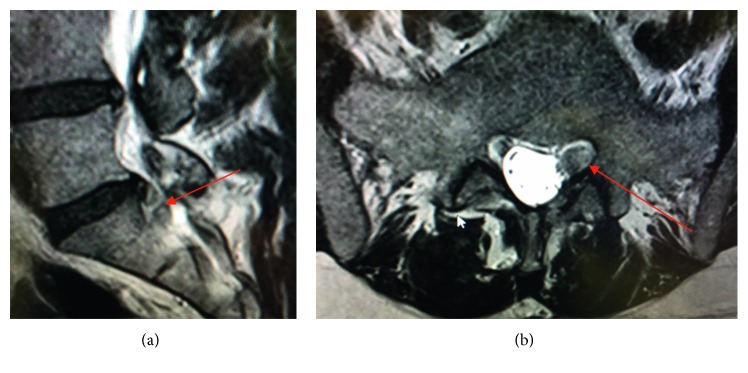
Lumbar MRI of case 3 with left-side S1 compression.

A 50-year-old male suffered from dorsal leg pain (VAS 7) since 8 months. Paresthesia and weakness of foot flexion (M3) were observed. The straight leg test was positive at 40°. No improvement was seen after conservative treatment. The MRI showed a free sequester caudally dislocated to the left side of the canal sacral (Figure 15; red arrow). By surgery, we made additional chromodiscography of L5-S1 disc. The caudally migrated sequester was visible by contrast dyeing (Figure 6, red arrow). We removed 2 pieces of disc sequester through the bone hole (Figure 9), and the S1 root was visually seen under endoscopic view (Figure 9).

After surgery, the leg pain disappeared. Only low back pain was still present. Foot flexion was improved to M4 and recovered completely after 8 weeks. The postoperative CT examination showed the access way through the S1 pedicle and the bone hole (Figure 10, red arrow).

## 3. Results

In all the 3 cases, we could remove the sequester through the bone hole and achieve a very rapid recovery. No fracture of the pedicle was found. One day after surgery, the pain score of leg pain was rapidly reduced to 1.5.

The neurology improved in 2 cases and completely recovered after 2 months.

## 4. Discussion

The newly developed technique of transpedicular endoscopic surgery [16–20] is also suitable for removing highly downmigrated L5-S1 disc herniation. This paper is the first description of the transpedicular endoscopic technique in highly downmigrated L5-S1 disc herniation.

If the access is exactly to the sequester through the S1 pedicle, we could decompress the S1 root and find a very rapid recovery. No complications were observed, especially no fracture of the S1 pedicle (sacrum).

The results of the new technique of three cases improve the endoscopic surgery of sequestered disc herniation at level L5-S1. Together with transforaminal and interlaminar endoscopic techniques, it is possible to use endoscopy for all kinds of disc herniation.

Many studies with a large number of cases are necessary, and comparable prospective studies should be performed to find the best technique.

Transpedicular endoscopy should be performed by very experienced endoscopic surgeons. If we enter the spinal canal by drilling the medial wall of the pedicle, it is possible to hit the root. So, it is safer to do this kind of surgery in analgosedation with local anesthesia. We developed the transpedicular technique in the last five years. In contrast to our first paper [16], we changed the technique. The access to the pedicle is in the most cases parallel and dorsal to the transverse process. We agree to Quillo-Olvera et al. [20] that the transpedicular accessway should be made dorsally of the transverse process. Only in cases with lumbar scoliosis and rotation of vertebra, it is useful to enter the pedicle anterior of the transverse process. The technique for the access to S1 pedicle differs from that of the other levels. The access way is very oblique from the iliac crest like in the transforaminal technique for L5-S1 [4], but caudally to the superior articular process and more dorsal into the S1 pedicle. There exist some gender differences in the anatomy of the sacrum and ilium. Because of the high iliac crest, it is more difficult to perform the technique in men than in women. The size of the S1 pedicle differs between the genders, but the pedicle S1 is part of the sacrum and mostly large enough to make a hole through. The height of the posterior pedicle is 19.74 mm on average in men and 18.21 mm in women [23]. In every case, we prefer the use of the bone shaver to improve the access, but at the beginning of making a bone hole, we use the Jamshidi needle followed by drills or reamers. Contraindications for the transpedicular technique included disc herniations without caudal sequestration and severe malformations of the sacrum.

A relative contraindication is the highly downmigrated medial disc L5-S1 herniation.

## 6. Conclusions

The new technique of transpedicular endoscopic surgery for highly downmigrated disc herniation of level L5-S1 showed excellent results in a small group of 3 cases. Further studies with a larger number of cases and prospective studies comparing interlaminar and transforaminal endoscopic surgery should be performed.

## Figures and Tables

**Table 1:** 

Name	Sex	Age	Level/side	VAS leg pain before operation	Neurology before operation	VAS leg pain after operation	Neurology after operation
Z. M.	Male	31	L5-S1 left side	8	Foot flexion M3-4	1	Foot flexion M4
S. W.	Male	56	L5-S1 left side	8-9	Numbness S1 leg	2	Numbness reduced to the foot
M. G.	Male	50	L5-S1 left side	7	Foot flexion M3	1-2	Foot flexion M4

## References

[1] Hijikata S., Yamagishi M., Nakayama T. (1975). Percutaneous nucleotomy: a new treatment method for lumbar disc herniation. *Journal of Toden Hospital*.

[2] Kambin P., Gellman H. (1983). Percutaneous lateral discectomy of lumbar spine: a preliminary report. *Clinical Orthopaedics and Related Research*.

[3] Yeung A., Yeung C. (2003). Advances in endoscopic disc and spine surgery: foraminal approach. *Surgical Technology International*.

[4] Schubert M., Hoogland T. (2005). Die transforaminale endoskopische nukleotomie mit foraminoplastik bei lumbalen bandscheibenvorfällen. *Operative Orthopädie und Traumatologie*.

[5] Ruetten S., Komp M., Merk H., Godolias G. (2009). Surgical treatment for lumbar lateral recess stenosis with the full-endoscopic interlaminar approach versus conventional microsurgical technique: a prospective, randomized, controlled study. *Journal of Neurosurgery: Spine*.

[6] Kim H. S., Paudel B., Jang J. S., Lee K., Oh S. H., Jang I. T. (2018). Percutaneous endoscopic lumbar discectomy for all types of lumbar disc herniations (LDH) including severely difficult and extremely difficult LDH cases. *Pain Physician*.

[7] Zekaj E., Menghetti S.-H., Saleh C. (2016). Contralateral interlaminar approach for intraforaminal lumbar degenerative disease with special emphasis on L5-S1 level: a technical note. *Surgical Neurology International*.

[8] Choi K.-C., Kim J.-S., Ryu K.-S., Kang B. U., Ahn Y., Lee S.-H. (2013). Percutaneous endoscopic lumbar discectomy for L5–S1 disc herniation: transforaminal versus interlaminar approach. *Pain Physician*.

[9] Lee S., Kim S.-K., Lee S.-H. (2006). Percutaneous endoscopic lumbar discectomy for migrated disc herniation: classification of disc migration and surgical approaches. *European Spine Journal*.

[10] Yeom K.-S., Choi Y.-S. (2011). Full endoscopic contralateral transforaminal discectomy for distally migrated lumbar disc herniation. *Journal of Orthopaedic Science*.

[11] Ahn Y., Jang I.-T., Kim W.-K. (2016). Transforaminal percutaneous endoscopic lumbar discectomy for very high-grade migrated disc herniation. *Clinical Neurology and Neurosurgery*.

[12] Choi G., Lee S. H., Lokhande P. (2008). Percutaneous endoscopic approach for highly migrated intracanal disc herniations by foraminoplastic technique using rigid working channel endoscope. *Spine*.

[13] Hu Q.-F., Pan H., Fang Y.-Y., Jia G.-Y. (2017). Percutaneous endoscopic lumbar discectomy for high-grade down-migrated disc using a trans-facet process and pedicle-complex approach: a technical case series. *European Spine Journal*.

[14] Sairyo K., Chikawa T., Nagamachi A. (2018). State-of-the-art transforaminal percutaneous endoscopic lumbar surgery under local anesthesia: discectomy, foraminoplasty, and ventral facetectomy. *Journal of Orthopaedic Science*.

[15] Liu C., Chu A. E., Yong H.-C., Chen L., Deng Z.-L. (2017). Percutaneous endoscopic lumbar discectomy for highly migrated lumbar disc herniation. *Pain Physician*.

[16] Krzok G., Telfeian A. E., Wagner R., Iprenburg M. (2016). Transpedicular lumbar endoscopic surgery for highly migrated disk extrusions: preliminary series and surgical technique. *World Neurosurgery*.

[17] Krzok G., Telfeian E. A., Wagner R., Iprenburg M. (2016). Transpedicular endoscopic surgery for spinal synovial cyst—new technique. *Journal of Spine Surgery*.

[18] Krzok G. Transpedicular endoscopic surgery.

[19] Jho H.-D. (1999). Endoscopic transpedicular thoracic discectomy. *Neurosurgery Focus*.

[20] Uniyal P., Choi G., Khedkkar B. (2016). Percutaneous transpedicular lumbar endoscopy: a case report. *International Journal of Spine Surgery*.

[21] Quillo-Olvera J., Akbary K., Kim J.-S. (2018). Percutaneous endoscopic transpedicular approach for high-grade down-migrated lumbar disc herniations. *Acta Neurochirurgica*.

[22] Choi G., Kim J.-S., Lokhande P., Lee S.-H. (2009). Percutaneous endoscopic lumbar discectomy by transiliac approach: a case report. *Spine*.

[23] Sinha M. B., Rathore M., Trivedi S., Siddiqui A. U. (2013). Morphometry of the first pedicle of sacrum and its clinical relevance. *International Journal of Healthcare & Biomedical Research*.

